# Xiasangju Processing Residues Improve Production Performance and Modulate Intestinal Inflammation and Gut Microbiota in Laying Hens

**DOI:** 10.3390/ani16121841

**Published:** 2026-06-15

**Authors:** Yiwei Jin, Lu Liu, Wei Wang, Pingping Li, Panpan Shi, Wei Liu, Peng Huang

**Affiliations:** 1 Hunan Key Laboratory of Chinese Veterinary Medicine, College of Veterinary Medicine, Hunan Agricultural University, Changsha 410128, China; jin_yiwei@stu.hunau.edu.cn (Y.J.); ll1234567811@163.com (L.L.); wangwei_2023@stu.hunau.edu.cn (W.W.); 488162468@stu.hunau.edu.cn (P.L.); 1140110690@stu.hunau.edu.cn (P.S.); liuwei618@hunau.edu.cn (W.L.); 2 Yuelushan Laboratory, Changsha 410128, China; 3 College of Bioscience and Biotechnology, Hunan Agricultural University, Changsha 410128, China

**Keywords:** Xiasangju processing by-products, late-laying hens, egg quality, COX-2, gut microbiota

## Abstract

With the increasing demand for sustainable animal production, the utilization of agricultural and processing by-products as functional feed ingredients has gained growing attention. However, the application of Xiasangju processing residues, a by-product generated during the industrial processing of the traditional Chinese herbal formula Xiasangju, in poultry nutrition remains poorly understood. This study aimed to evaluate the nutritional composition, bioactive components, and potential functional effects of Xiasangju processing by-products as a dietary supplement for late-laying hens. Chemical composition was characterized using LC-MS and conventional nutritional analysis, while potential anti-inflammatory mechanisms were predicted via network pharmacology. A total of 288 Jingfen laying hens (55 weeks old) were randomly assigned to four dietary treatments supplemented with 0, 0.5%, 1.0%, or 1.5% Xiasangju processing by-products for 56 days. Results showed that 11 major active compounds were identified, including relatively high levels of linarin and rosmarinic acid, along with abundant crude protein, fiber, minerals, and amino acids. Dietary supplementation at 1.5% was associated with higher egg production rate, egg weight, and yolk color without obvious adverse effects on organ indices or serum biochemical parameters. This treatment was also associated with lower inflammatory gene expression, including *IL-6* and *COX-2* in the ileum and *NF-κB*, *IL-6*, *COX-2*, and *TNF-α* in the cecum. No significant effects were observed on antioxidant status in yolk or liver, or intestinal morphology. Integrated analyses using network pharmacology, molecular docking, and in vitro COX-2 inhibition suggested that anti-inflammatory-related responses may involve COX-2-related pathways, while 16S rRNA sequencing indicated changes in gut microbiota composition. In conclusion, Xiasangju processing by-products may serve as a potential functional feed ingredient for late-laying hens, with 1.5% inclusion showing favorable overall performance under the conditions of this study. These effects may be associated with the combined contribution of residual nutrients and bioactive compounds in the residues.

## Simple Summary

Xiasangju processing residues are plant-based by-products generated during the production of traditional Chinese medicine formulations, which may still contain polyphenols, flavonoids, and certain nutrients. This study evaluated the potential effects of incorporating this processingby-products into the diets of laying hens during the late laying period. The results indicated that Xiasangju processing residues contain active compounds such as linarin, rosmarinic acid, and chlorogenic acids; a 1.5% inclusion level was associated with favorable results in terms of egg production rate, egg weight, and yolk color. Further analysis suggests that its effects may be related to COX-2-mediated regulation of intestinal inflammation and changes in the gut microbiota composition. This study provides insights into the resource utilization of by-products from traditional Chinese medicine processing in layer chicken production.

## 1. Introduction

Against the background of reduced antibiotic use in animal feed, phytobiotics and plant-derived feed additives have attracted increasing attention in poultry nutrition because they may support gut integrity, antioxidant status, immune function, and microbial balance [1]. Compounds commonly found in plant-based ingredients, such as flavonoids, polyphenols, polysaccharides and terpenoids, are associated with antioxidant, anti-inflammatory and immunomodulatory effects [2]. Among these, polyphenolic compounds such as chlorogenic acid, caffeic acid and rosmarinic acid contribute to the body’s protective mechanisms by regulating the redox balance and the release of inflammatory mediators [3]. Certain plant extracts can also influence oxidative stress and gut homeostasis via pathways such as the ‘gut microbiota–gut–liver axis’ [4]. Meanwhile, traditional Chinese medicine processing residues are increasingly regarded as recyclable biomass resources, because they may retain nutrients and bioactive compounds and can be valorized through feed, bioactive-compound recovery, biochar, or other resource-utilization pathways [5]. The resource recovery of Chinese herbal medicine residues not only alleviates the pressure on waste management but also expands the range of unconventional feedstock sources [6]. Methods such as fermentation or recycling can improve the nutritional bioavailability and functional metabolite profiles of by-products from traditional Chinese medicine [7]. Animal studies have further demonstrated that by-products from the processing of traditional Chinese medicine show some potential for improving intestinal morphology, antioxidant capacity and inflammatory status [8,9].

Xiasangju is a traditional Chinese herbal formula composed of *Prunella vulgaris* L., *Morus alba* L., and *Chrysanthemum indicum* L. [10]. *Prunella vulgaris* contains active compounds such as rosmarinic acid, caffeic acid, luteolin and rutin, and exhibits biological activities including anti-inflammatory, antioxidant and immunomodulatory effects [11]. Mulberry leaves are rich in compounds such as chlorogenic acid, rutin, quercetin and 1-deoxynojirimycin, and have attracted attention for their role in regulating carbohydrate and lipid metabolism and for their antioxidant properties [12]. Wild chrysanthemum contains phenolic acids and flavonoids such as chlorogenic acid, linarin, luteolin and acacetin, and exhibits certain antibacterial and anti-inflammatory properties [13]. Consequently, although Xiasangju processing residues undergo industrial extraction, they may still contain residual phenolic acids and flavonoids with functional significance; it is therefore worth further evaluating their potential for use as animal feed.

In the late laying phase, laying hens often experience a decline in egg production, fluctuations in egg quality and an increased metabolic burden [14]. Calcium and phosphorus supply, eggshell formation and bone metabolism also influence the egg-laying performance and eggshell quality of laying hens in the later stages [15]. The composition of the gut microbiota is closely linked to egg quality and safety, and can serve as a key target for nutritional regulation in laying hens [16]. As the birds age, the barrier function of the intestinal mucosa in laying hens may decline during the middle and late stages of the laying cycle, further highlighting the need for regulation of intestinal homeostasis [17]. In recent years, phytogenic feed additives have been evaluated not only for their effects on laying performance and egg quality, but also for their potential roles in intestinal barrier function, immune homeostasis, and gut microbiota regulation [18]. Research into the application of plant-based additives in poultry nutrition also suggests that they may influence host health by modulating the gut microbiota and local inflammatory responses [19]. Previous studies have shown that mulberry leaf extract can improve egg quality and regulate lipid metabolism [20]; Compound Chinese herbal residues have also shown potential for improving production performance, egg quality and intestinal barrier integrity in laying hens during the late laying phase [21]. However, there is still limited research on the use of Xiasangju processing residues in laying hens during the late laying phase, and their residual active components, nutritional value as a feed ingredient, and potential effects on intestinal inflammation and gut microbiota regulation remain unclear.

Accordingly, this study first employed LC-MS and conventional nutrient analysis to identify the major bioactive components and nutritional characteristics of Xiasangju processing residues, and combined this with network pharmacology to predict their potential anti-inflammatory targets; Subsequently, using 55-week-old Jingfen laying hens as test subjects, four supplementation levels (0%, 0.5%, 1.0% and 1.5%) were established to evaluate their effects on production performance, egg quality, serum biochemistry, antioxidant levels, intestinal morphology, inflammation-related gene expression, COX-2 activity and gut microbiota composition.

The aim of this study is to evaluate the potential of Xiasangju processing residues as a dietary supplement for laying hens in the late laying phase, and to preliminarily explore its possible associations with COX-2-related inflammatory responses and changes in gut microbiota composition.

## 2. Materials and Methods

### 2.1. Test Materials and Major Reagents

The Xiasangju processing residues used in this study were provided by Guangzhou Baiyunshan Xingqun (Pharmaceutical) Co., Ltd. (Guangzhou, China). These residues were generated during the industrial production of Xiasangju granules, a traditional Chinese herbal formula composed mainly of *Prunella vulgaris* L., *Morus alba* L., and *Chrysanthemum indicum* L. According to the available production records, the raw materials were prepared at a weight ratio of 500:175:80 for *Prunella vulgaris* L., *Morus alba* L., and *Chrysanthemum indicum* L., respectively. Briefly, *Chrysanthemum indicum* L. was macerated with 95% ethanol at 30 °C for 16 h at a solid-to-liquid ratio of 1:2 to obtain residue 1. *Prunella vulgaris* L. and *Morus alba* L. were decocted with six volumes of water twice, each for 1.5 h, to obtain residue 2. Residues 1 and 2 were then combined, dried, ground, and passed through a 60-mesh sieve to obtain the Xiasangju processing residue powder. To improve the representativeness of the samples and minimize the impact of variations in a single batch of raw materials on the test results, a total of 15 samples from different production batches were collected, with each sample weighing between 500 and 1000 g. After each batch of samples was thoroughly mixed, one portion was used for subsequent active ingredient testing and routine nutritional analysis, while the other portion was set aside as feed material for laying hen feeding trials.

The methanol and acetonitrile used in the LC-MS analysis were HPLC grade and were purchased from Merck KGaA (Darmstadt, Germany); formic acid was LC-MS grade and was purchased from Sigma-Aldrich (St. Louis, MO, USA). The main instruments used in the experiments included an ultra-high-performance liquid chromatography–triple quadrupole mass spectrometry system (Agilent Technologies, Santa Clara, CA, USA), a high-performance liquid chromatography system (Agilent Technologies, Santa Clara, CA, USA), an ultrasonic extractor (KQ-500DE, Kunshan Ultrasonic Instruments Co., Ltd., Kunshan, China), a high-speed centrifuge (5810 R, Eppendorf SE, Hamburg, Germany), an analytical balance (CPA series, Sartorius AG, Göttingen, Germany), and a Thermo Barnstead ultrapure water system (Thermo Fisher Scientific, Waltham, MA, USA). The reference standards, including neochlorogenic acid, chlorogenic acid, cryptochlorogenic acid, isochlorogenic acid A, isochlorogenic acid B, isochlorogenic acid C, caffeic acid, rosmarinic acid, acacetin, linarin, and isorosmarinic acid glycoside, were purchased from Aladdin Chemical Co., Ltd. (Shanghai, China), and their purity met the requirements for both qualitative and quantitative analysis.

### 2.2. Sample Preparation and Compositional Analysis of Xiasangju Processing Residues

Before compositional analysis, the Xiasangju processing residue powder was further mixed thoroughly and passed through a 60-mesh sieve. A 100 g sample from each batch was set aside for storage, and the remainder was used for subsequent extraction and testing. To ensure the stability of methodological evaluation and component identification, 100 g was weighed from each of the 15 batches of sieved samples, thoroughly mixed, and used to prepare quality control samples.

To prepare the test solution, 0.50 g of Xiasangju processing residue powder was accurately weighed, placed in a 50 mL centrifuge tube, mixed with 25.0 mL of methanol, weighed, and subjected to ultrasonic extraction for 30 min. After the extract had cooled to room temperature, solvent loss was compensated with methanol to the original mass. The solution was then mixed thoroughly and filtered through a 0.22 μm organic microporous membrane to obtain the test solution for LC-MS analysis.

To improve the accuracy of identifying chemical constituents in Xiasangju processing residues, this study compiled the major chemical constituents previously reported in *Prunella vulgaris* L, *Morus alba* L, and *Chrysanthemum indicum* L. from databases such as CNKI and Web of Science, and used this information to establish a mass spectrometry database. The relevant chemical constituent information is provided in Supplementary Table S1. This database was used for the rapid matching, screening, and annotation of candidate constituents in subsequent samples.

LC-MS analysis was performed using an Agilent Eclipse XDB-C18 column (Agilent Technologies, Santa Clara, CA, USA) for separation, with a 0.1% formic acid aqueous solution as mobile phase A and acetonitrile as mobile phase B, employing gradient elution. The flow rate was 0.30 mL/min, the column temperature was 35 °C, and the injection volume was 1 μL. Mass spectrometry utilized an electrospray ionization (ESI) source to acquire high-resolution first-order mass spectrometry data in negative ion mode, with a scan range of *m*/*z* 100–1000. Second-order mass spectrometry employed the target MS/MS mode, with collision energies optimized within the range of 5–45 eV based on the structural characteristics of the target compounds. Data acquisition was performed using Agilent MassHunter software (Version B.05.00, Agilent Technologies, Santa Clara, CA, USA); the subsequent data screening and annotation methods are described in Section 2.11.

To evaluate the reliability of the assay method, this study conducted a methodological assessment based on four criteria: specificity, reproducibility, precision, and stability. Specificity was confirmed based on the retention time, exact molecular weight, and extraction ion chromatographic peaks of the standard; reproducibility was assessed by analyzing six parallel preparations of the same quality control sample; precision was evaluated by injecting the same test solution six times in succession; and stability was assessed by analyzing the test solution after storage at room temperature for 0, 6, 12, 24, 36, and 48 h.

To establish the calibration curves, 11 target compound standards were weighed separately to prepare single-standard stock solutions and mixed standard working solutions. After analysis by LC-MS, a linear regression equation was established with the mass concentration of the target compound on the *x*-axis and the peak area of the extracted ion chromatogram on the *y*-axis, and the concentration of the target compound in the sample was calculated based on this equation. See Supplementary Table S2 for the standard curves and correlation coefficients of the 11 target compounds.

The routine nutritional components of Xiasangju processing residues were determined in accordance with national standard methods: crude protein was determined using the Kjeldahl method; crude fat was determined using the Soxhlet extraction or filter bag method; crude ash was determined using the ashing method; amino acid composition was determined in accordance with the method for determining amino acids in feed; and total energy was determined using the oxygen bomb calorimetry method.

### 2.3. Network Pharmacology Analysis

Based on LC-MS identification and quantitative analysis, 11 major active compounds identified in Xiasangju processing residues were selected as targets for network pharmacology analysis. First, information such as the compound name, molecular formula, relative molecular mass, and canonical SMILES was retrieved from the PubChem database, and the structural information was manually verified. For active compound-related target selection, entries were retained only when they had clear compound–target correspondence, available gene names, and could be standardized using the UniProt database, with priority given to Gallus gallus-related target information. Duplicated, invalid, and non-standardized targets were removed.

Targets related to antioxidant and anti-inflammatory functions were identified using the keywords “antioxidant,” “oxidative stress,” “anti-inflammatory,” and “inflammation” through searches of databases such as GeneCards, OMIM, DisGeNET, and TTD, followed by merging, deduplication, and standardization. By intersecting the potential targets of the active ingredient with those related to antioxidant and anti-inflammatory effects, we identified common targets.

The bioactive compounds and common targets were imported into Cytoscape (version 3.10.3) to construct a “bioactive compound–common target” network, and topological parameters such as degree, betweenness centrality, and closeness centrality were calculated. The common targets were further imported into the STRING database to construct a PPI network, with the species restricted to *Gallus gallus*, and the cytoHubba plugin was used to identify core targets. GO and KEGG pathway enrichment analyses were performed using the Metascape platform, with a significance threshold of *p* < 0.05 or a corrected Q < 0.05.

### 2.4. Animal Experiment Design and Husbandry

All animal procedures were approved by the Biomedical Research Ethics Committee of Hunan Agricultural University (Approval No.: LSK2023-161). The experiment was conducted in accordance with institutional guidelines for animal welfare and scientific ethics. All procedures, including husbandry, blood collection, euthanasia, and sample collection, were performed by trained personnel to minimize stress and discomfort to the birds.

The animal experiments were conducted at the Xingxin Layer Farm in Anxiang County, Changde City, Hunan Province. A total of 288 healthy 55-week-old Jingfen laying hens with similar egg production rates were selected. Using a single-factor completely randomized design, the hens were randomly divided into 4 groups, with 8 replicates per group and 9 hens per replicate. The control group was fed a basal diet, the composition and nutritional levels of which are shown in Table 1. The low-, medium-, and high-dose groups were fed basal diets supplemented with 0.5%, 1.0%, and 1.5% of Xiasangju processing residues, respectively, and were designated XSJ-L, XSJ-M, and XSJ-H. The basal diet formulation was recorded and is shown in Table 1. Xiasangju processing residues were added to the basal diet at the above inclusion levels according to the original feeding design. However, complete nutrient-balanced formulations of the final experimental diets, detailed formulation software outputs, and nutrient matrix values for all treatment diets were not available from the original experimental records. Therefore, the present study could not confirm that all experimental diets were fully isocaloric, isonitrogenous, or balanced for amino acids, calcium, and phosphorus. The basal diet was a corn-soybean meal diet formulated in accordance with the nutritional requirements for laying hens specified in the “Standards for Poultry Rearing (NY/T 33—2004).”

The experimental laying hens were housed in three-tiered cage systems, with three hens per cage, and partitioned into separate replicate units. During the experiment, the hens had free access to feed and water, were fed once daily at 6:30 a.m. and 2:30 p.m., and were kept under a standardized 16 h light cycle. Eggs were collected daily at 3:00 p.m., and the number of eggs laid, egg weight, and number of cracked, soft-shelled, and malformed eggs were recorded for each replicate. Rearing conditions were kept consistent across all treatment groups, and the vaccination schedule followed standard procedures for layer farms. The formal trial period lasted 56 days, during which the flock’s overall condition and feed intake were observed daily.

### 2.5. Sample Collection

After the experiment, the laying hens were fasted for 12 h. Eight hens were randomly selected from each group for sampling. After blood was collected from the subclavian vein, the blood sample was placed in a 10 mL centrifuge tube, allowed to stand at room temperature for 2 h, and then centrifuged at 3000 rpm at 4 °C for 10 min. After separation, the serum was stored on dry ice for subsequent serum biochemical testing. After slaughter, the liver, spleen, ovaries, and fallopian tubes were collected in that order, weighed, and used to calculate the organ index. Liver tissue was wrapped in aluminum foil and flash-frozen in liquid nitrogen for antioxidant analysis. After dissecting the duodenum, jejunum, ileum, and cecum, the contents were flushed out with pre-chilled saline. Approximately 2 cm segments of each were placed in 4% paraformaldehyde for fixation, to be used for histological examination. Separate samples of ileum and cecum tissue were placed in cryovials, rapidly frozen in liquid nitrogen, and stored at −80 °C for the detection of inflammation-related gene expression.

Samples for gut microbiota sequencing were collected at the end of the 8-week trial. Six laying hens were randomly selected from each group, and chyme samples were collected from the foregut and hindgut, respectively. The foregut chyme was prepared by pooling the contents from the duodenum, jejunum, and ileum of the same bird, while the hindgut chyme was prepared by pooling the contents from the cecum and rectum of the same bird. Foregut and hindgut samples were treated as two separate sample types and were homogenized separately under sterile conditions. The foregut and hindgut contents were not mixed with each other before DNA extraction. Immediately after collection and homogenization, the samples were rapidly frozen in liquid nitrogen and transferred to −80 °C for storage.

### 2.6. Measurement of Production Performance, Organ Indices, and Egg Quality

During the experiment, egg production, total egg weight, and the number of cracked, soft-shelled, and malformed eggs were recorded daily for each replicate, and feed intake was tallied every two weeks. The egg production rate, average egg weight, cracked, soft-shelled, and malformed egg rate, average daily feed intake, and feed-to-egg ratio were calculated based on the recorded data. The egg production rate is the percentage of total eggs laid relative to the number of hens; the average egg weight is the ratio of total egg weight to the total number of eggs laid; the cracked, soft-shelled, and malformed egg rate is the percentage of cracked, soft-shelled, and malformed eggs relative to the total number of eggs laid; the average daily feed intake is calculated based on feed consumption, the number of hens, and the number of days in the trial; the feed-to-egg ratio is the ratio of feed consumption to total egg weight.

After blood collection, the liver, spleen, ovaries, and fallopian tubes were quickly removed, blotted dry, and weighed. Organ indices were calculated as the percentage of organ weight relative to body weight, including the liver index, spleen index, ovarian index, and fallopian tube index.

One day before the end of the experiment, eggs were randomly selected from each group for egg quality assessment. Egg weight was measured using an electronic balance (CPA series, Sartorius AG, Göttingen, Germany); the horizontal and vertical diameters were measured using an electronic digital vernier caliper (Mitutoyo Corporation, Kawasaki, Japan), and the egg shape index was calculated; eggshell strength was measured using an eggshell strength tester (Egg Force Reader, ORKA Food Technology Ltd., Ramat HaSharon, Israel); after breaking the eggs, yolk color, albumen height, and Haugh units were determined using an Egg Analyzer^®^ (ORKA Food Technology Ltd., Ramat HaSharon, Israel); the thickness of the sharp end, blunt end, and middle of the eggshell was measured separately using an Eggshell Thickness Gauge (ORKA Food Technology Ltd., Ramat HaSharon, Israel), and the average was taken as the eggshell thickness; yolk height and diameter were measured using an electronic digital vernier caliper, and the yolk index was calculated; after removing the albumen and chalazae from the yolk surface, the yolk was weighed, and the yolk percentage was calculated.

### 2.7. Serum Biochemistry and Antioxidant Marker Testing

Serum parameters, including IgM, IgG, IgA, serum phosphorus, serum calcium, AST, ALT, ALP, ALB, GLU, TC, TG, HDL-C, and LDL-C, were measured using a Kehua fully automated biochemical analyzer (Shanghai Kehua Bio-engineering Co., Ltd., Shanghai, China). The relevant test kits were purchased from Shanghai Kehua Bio-engineering Co., Ltd. (Shanghai, China). Antioxidant parameters in liver and egg yolk were measured according to the instructions provided with the commercial kits from Beijing Box Biotech Co., Ltd. (Beijing, China). The parameters measured included total antioxidant capacity, superoxide dismutase activity, and malondialdehyde content.

### 2.8. Morphological Examination of the Intestine and Detection of Inflammation-Related Gene Expression

Tissues from the duodenum, jejunum, and ileum, fixed with 4% paraformaldehyde, were prepared as paraffin sections using standard methods and stained with hematoxylin and eosin (HE). After staining, the tissue structure was examined under an optical microscope. The villus height and crypt depth of each intestinal segment were measured using CaseViewer 2.4.0 image analysis software, and the ratio of villus height to crypt depth was calculated.

Tissue samples from the ileum and cecum were used to detect the expression of inflammation-related genes. An appropriate amount of tissue was mixed with Trizol lysis buffer and homogenized thoroughly. Total RNA was obtained after chloroform extraction, isopropanol precipitation, and washing with 75% DEPC-treated ethanol. RNA concentration and purity were determined using a nucleic acid analyzer; samples with an OD260/280 ratio between 1.8 and 2.0 were used for subsequent reverse transcription. cDNA synthesis was performed using the RT Mix Kit with gDNA Clean for qPCR (Accurate Biotechnology (Hunan) Co., Ltd., Changsha, China). Real-time quantitative PCR was performed using the SYBR Green Pro Taq HS qPCR Kit, with *β-actin* as the internal control, to determine the relative expression levels of target genes including *IL-1β*, *IL-4*, *IL-6*, *IL-10*, *NF-κB*, COX-2, and *TNF-α*.

### 2.9. Molecular Docking and In Vitro COX-2 Inhibition Assays

Based on the LC-MS identification and quantitative analysis, the 11 representative active compounds detected in Xiasangju processing residues were selected for molecular docking. These compounds were selected because they were identified as residual phenolic acids or flavonoids in Xiasangju processing residues and were included in the compound–target network analysis. COX-2 was selected as the docking protein because PTGS2, which encodes COX-2, was identified as a potential inflammation-related target in the network pharmacology analysis and was also related to the subsequent intestinal inflammatory gene expression results. For the in vitro COX-2 inhibition assay, the total extract of Xiasangju processing residues and three representative compounds, namely neochlorogenic acid, linarin, and rosmarinic acid, were selected based on their detection in LC-MS analysis, compound category, and relevance to COX-2-related inflammatory responses. The crystal structure of the COX-2 protein was obtained from the RCSB PDB, and the 2D structures of the 11 active compounds were obtained from PubChem. Molecular docking was performed using AutoDockTools (version 1.5.7), and binding affinity was evaluated based on binding energy. A COX-2 inhibitor screening kit (Beyotime Biotechnology, Shanghai, China) was used to assess the COX-2 inhibitory activity of the total extract of Xiasangju processing residues, as well as neochlorogenic acid, linarin, and rosmarinic acid. Celecoxib served as the positive control, with three replicates for each treatment.

### 2.10. Gut Microbiome Sequencing and Analysis

After the experiment, samples of chyme were collected from the foregut and hindgut of the laying hens, rapidly frozen in liquid nitrogen, and then transferred to −80 °C for storage. Total microbial DNA from the samples was extracted using the FastPure Stool DNA Isolation Kit (Shanghai Meiji Biotechnology Co., Ltd., Shanghai, China). DNA integrity was assessed by 1% agarose gel electrophoresis, and concentration and purity were determined using a NanoDrop 2000 spectrophotometer (Thermo Fisher Scientific, Waltham, MA, USA).

Using the extracted DNA as a template, the V3–V4 region of the bacterial 16S rRNA gene was amplified using the barcoded primers 338F (5′-ACTCCTACGGGAGGCAGCAG-3′) and 806R (5′-GGACTACHVGGGTWTCTAAT-3′). The PCR amplification program was as follows: 3 min pre-denaturation at 95 °C; followed by 27 cycles consisting of 30 s at 95 °C, 30 s at 55 °C, and 30 s at 72 °C; and a final 10 min extension at 72 °C. The amplification products were separated by 2% agarose gel electrophoresis, excised from the gel, and purified using a PCR Clean-Up Kit.

Sequencing libraries were constructed from qualified samples using the NEXTFLEX Rapid DNA-Seq Kit, and high-throughput sequencing was performed on the Illumina NextSeq 2000 platform (Illumina, San Diego, CA, USA). The sequencing data were analyzed using the Meiji Bioinformatics Cloud Platform, primarily focusing on α-diversity, OTU distribution, PCA analysis, species composition, and LEfSe differential microbiome analysis.

### 2.11. Statistical Analysis

The raw LC-MS data were processed using Agilent MassHunter Qualitative Software (Version B.05.00, Agilent Technologies, Santa Clara, CA, USA). The screening of sample compounds was performed using the molecular feature extraction function, and qualitative confirmation was achieved by combining this with Xiasangju’s theoretical mass spectrometry database of reported chemical constituents, high-resolution MS/MS fragment information, and the retention times of reference standards. Data on active ingredient content, general nutritional components, and amino acid composition were analyzed using Excel 2024, and results are expressed as mean ± standard deviation.

After preliminary organization of the network pharmacology data in Excel, the data were analyzed and visualized using R software (version 4.3.2) and Cytoscape (version 3.10.3). For animal experiment data, each replicate was treated as a statistical unit, and statistical analysis was performed using SPSS 23.0. One-way analysis of variance (ANOVA) was performed on production performance, organ indices, egg quality, serum biochemistry, and intestinal morphology data; when significant differences were observed, post hoc tests were conducted using the Tukey–Kramer method. Linear and quadratic effect analyses were further conducted on production performance, organ indices, and egg quality. For parameters comparing only the Control group and the XSJ-H group—such as inflammation-related gene expression, COX-2 in vitro inhibitory activity, and certain mechanistic validation data—an independent samples *t*-test was used.

Results from animal experiments and mechanism validation are expressed as mean ± standard error. *p* < 0.05 indicates a significant difference, and *p* < 0.01 indicates a highly significant difference. Figures were generated using GraphPad Prism 10.0. When comparing results to the Control group, * indicates *p* < 0.05, ** indicates *p* < 0.01, and *** indicates *p* < 0.001.

## 3. Results

### 3.1. Analysis of the Active Ingredients and Nutritional Composition of Xiasangju Processing Residues

#### 3.1.1. Validation of LC-MS Quantitative Methodology

To clarify the material composition of the processing residues from Xiasangju, this study first established an LC-MS method for qualitative and quantitative analysis. The results of the methodological evaluation showed that the repeatability and precision (RSD) of the peak response values for the 11 target compounds were all less than 5%. In the stability study, except for acacetin and isochlorogenic acid A, which had RSD values of less than 7%, the RSD values for all other compounds were less than 5%. This indicates that the method exhibits good repeatability, precision, and stability and is suitable for subsequent batch sample analysis. The detailed results of the methodological validation are presented in Supplementary Table S3.

#### 3.1.2. Ion Chromatogram of the Target Compound

**Figure 1:**
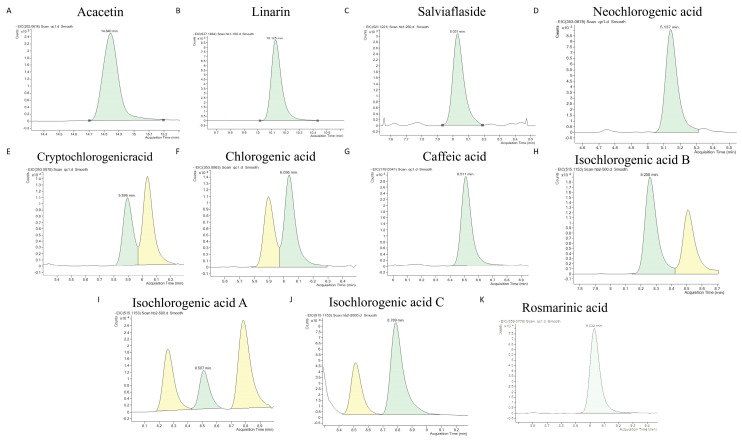
Extraction ion chromatogram (EIC) of target compounds from Xiasangju processing residues. (**A**–**K**) Extraction ion current chromatograms of neochlorogenic acid, chlorogenic acid, Cryptochlorogenic acid, isochlorogenic acid A, isochlorogenic acid B, isochlorogenic acid C, caffeic acid, Salviaflaside, Rosmarinic acid, Linarin, and Acacetin. The green and yellow shaded areas indicate software-generated peak integration regions, and the black squares indicate peak boundary markers generated during chromatographic peak integration.

To examine the chromatographic separation and mass spectrometric responses of the target compounds in samples of Xiasangju processing residues, this study generated characteristic ion chromatograms for 11 target compounds (Figure 1A–K). The results showed that distinct chromatographic peaks with clear shapes appeared at the corresponding retention times for each target compound, indicating that this method is capable of effectively distinguishing the major target components in Xiasangju processing residues.

#### 3.1.3. Identification of Major Chemical Components in Xiasangju Processing Residues by LC-MS

**Figure 2:**
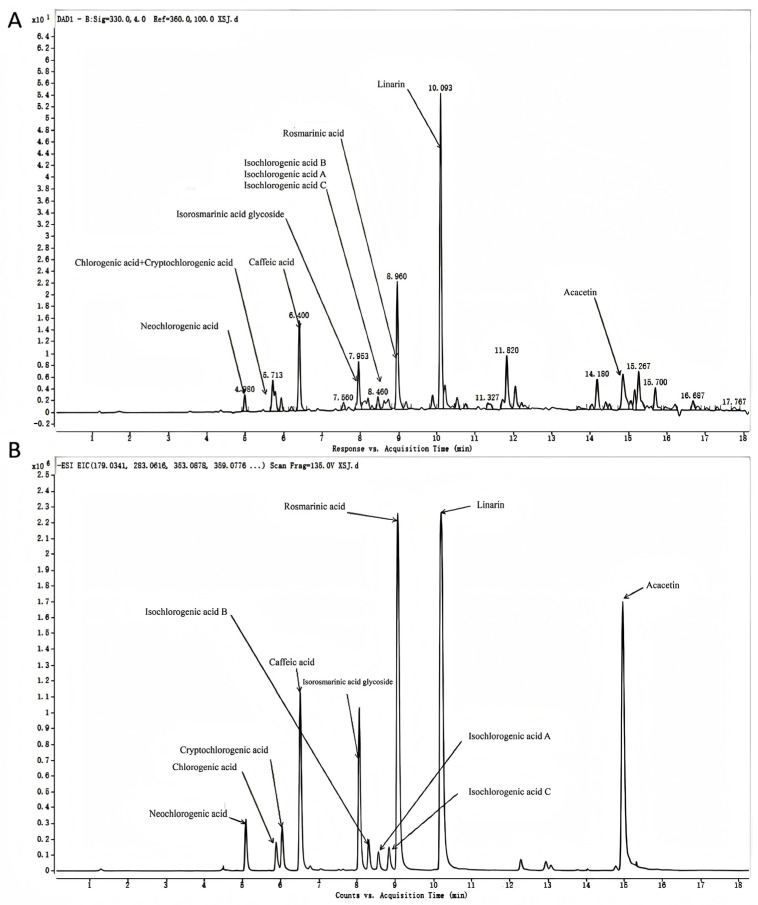
Results of LC-MS qualitative analysis of the chemical composition of Xiasangju processing residues. (**A**) LC-MS qualitative analysis of major target compounds in the Xiasangju processing residue sample; (**B**) Spectra of standard samples or controls, used to assist in confirming the retention times and mass spectrometric responses of target compounds in the sample.

Based on ion-polarization flow analysis, combined with high-resolution secondary mass spectrometry fragment data and retention times of standard compounds for qualitative comparison, 11 major active components were ultimately identified in the sample: neochlorogenic acid, chlorogenic acid, Cryptochlorogenic acid, isochlorogenic acid A, isochlorogenic acid B, isochlorogenic acid C, caffeic acid, Salviaflaside, rosmarinic acid, linarin, and acacetin. These results indicate that Xiasangju processing residues retain a certain amount of phenolic acids and flavonoids even after industrial extraction (Figure 2A,B).

#### 3.1.4. Content of 11 Active Compounds in Xiasangju Processing Residues

Quantitative results showed that the content of various components varied among different batches of samples. Linarin had the highest content, averaging 83.22 ± 5.62 μg/g; rosmarinic acid followed, at 25.92 ± 13.47 μg/g; the average contents of Salviaflaside, Acacetin, and caffeic acid were 10.68 ± 4.71, 10.18 ± 5.53, and 9.08 ± 4.75 μg/g, respectively. The total content of neochlorogenic acid, Cryptochlorogenic acid, chlorogenic acid, and isochlorogenic acid derivatives was relatively low; isochlorogenic acid B was not detected in some batches of samples. The results above indicate that Xiasangju processing residues retain certain active phenolic and flavonoid compounds even after industrial extraction. The complete analytical results for the 11 active compounds in each batch sample and the QC sample are presented in Supplementary Table S4, and the average content of the major components is shown in Table 2. The corrected standard deviation of linarin indicates relatively limited variation among the analyzed batches; nevertheless, the characterization of representative active compounds remains important for the quality control and feed application of Xiasangju processing residues.

#### 3.1.5. Typical Nutritional Composition of Xiasangju Processing Residues

The standard nutritional analysis results are shown in Table 3. The crude protein content of Xiasangju processing residues was 12.60 ± 1.49%, with an energy content of 18.39 ± 1.85 MJ/kg; the crude fat, crude fibre and crude ash contents were 7.52 ± 1.28%, 12.49 ± 1.52% and 13.17 ± 1.58% respectively. Amino acid analysis identified 17 amino acids, with a total amino acid content of 8.45%. Glutamic acid was the most abundant, followed by aspartic acid and leucine; the specific amino acid composition is shown in Supplementary Table S5. These results suggest that Xiasangju processing residues not only retain certain active phenolic and flavonoid compounds but also possess a certain level of basic nutritional value, providing a material basis for subsequent feeding trials in laying hens.

### 3.2. Analysis of Potential Antioxidant and Anti-Inflammatory Targets in Xiasangju Processing Residues

#### 3.2.1. Analysis of the Overlap Between Potential Targets of Active Ingredients and Targets Associated with Antioxidant and Anti-Inflammatory Effects

**Figure 3:**
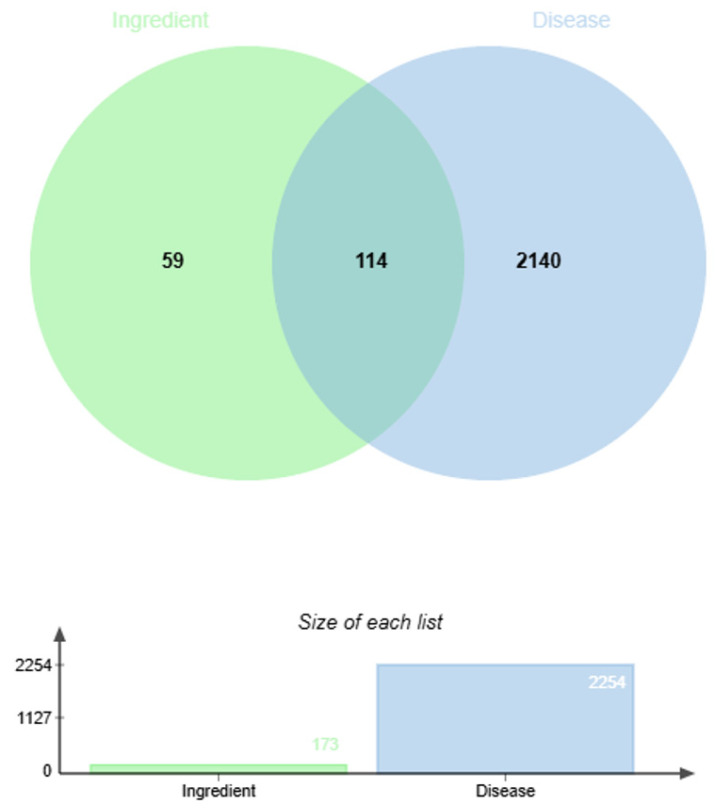
Venn diagram showing the potential targets of the major active components in Xiasangju processing residues and targets related to antioxidant and anti-inflammatory effects.

This study used 11 major bioactive compounds identified by LC-MS as the subjects of the network pharmacology analysis; the two-dimensional structures of the relevant compounds are shown in Supplementary Figure S1. The results of the target prediction analysis revealed that the 11 active compounds identified a total of 173 potential targets; after intersecting these with targets associated with antioxidant and anti-inflammatory diseases, 114 common targets were identified (Figure 3), suggesting that these targets may form the key basis for the antioxidant and anti-inflammatory effects of Xiasangju processing residues.

#### 3.2.2. Active Ingredients—Identification of Shared Target Networks and Core PPI Targets

**Figure 4:**
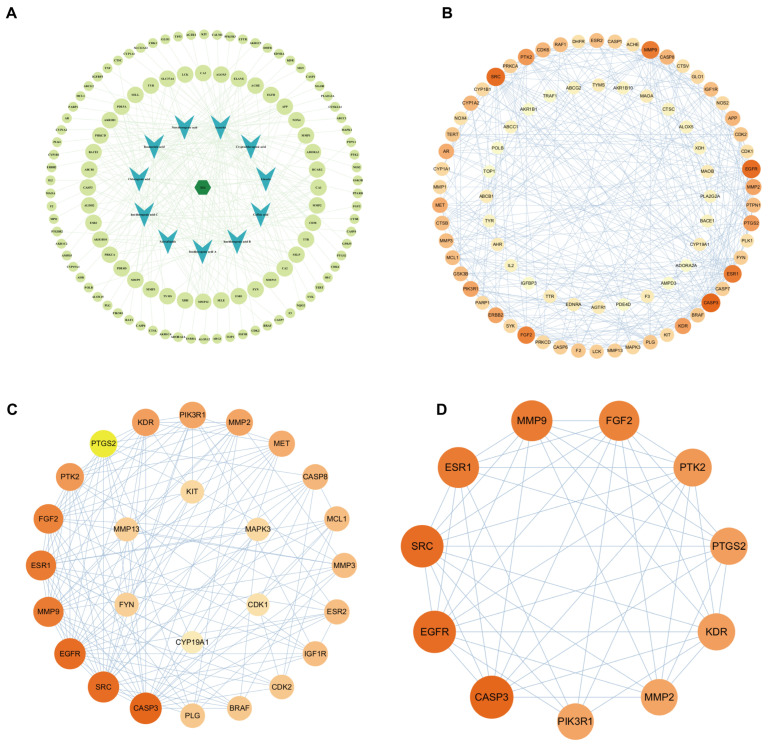
Network of bioactive compounds from Xiasangju processing residues and their common targets, and a diagram showing the selection of core PPI targets. (**A**) Network of bioactive compounds from Xiasangju processing residues and their common targets. (**B**) PPI interaction network of shared targets. (**C**) Network of key targets following further topological screening. (**D**) Final core target network identified through screening. Blue lines indicate interactions or associations between nodes in the network.

The compound–target network, PPI network, key-target network, and final core-target network are shown in Figure 4A–D.The “processing residues–active ingredients–targets” network and the PPI interaction network were further constructed. The results of the PPI network topology analysis indicate that *PTGS2*, *FGF2*, *MMP9*, *PTK2*, *ESR1*, *SRC*, *EGFR*, *CASP3*, *PIK3R1*, *MMP2* and *KDR* are core targets. Among these, *PTGS2* encodes COX-2, which is closely associated with the production of inflammatory mediators and arachidonic acid metabolism, suggesting that COX-2 may be one of the potential targets associated with inflammatory responses after Xiasangju processing residue supplementation.

#### 3.2.3. Enrichment Analysis of Shared Targets in GO Functions and KEGG Pathways

**Figure 5:**
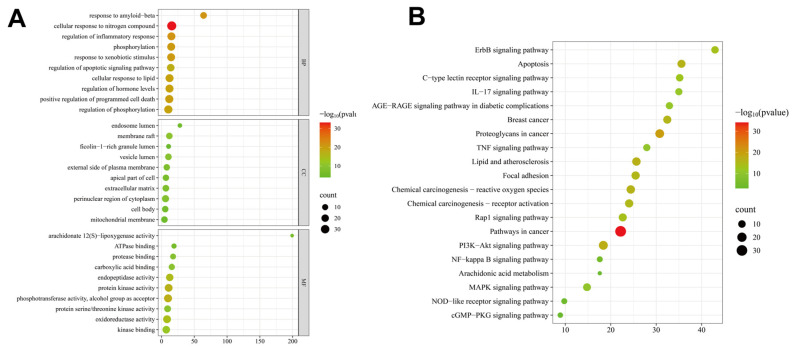
GO functional and KEGG pathway enrichment analyses of common targets of Xiasangju processing residues. (**A**) Results of the GO functional enrichment analysis of common targets. (**B**) Results of the KEGG pathway enrichment analysis of common targets.

GO enrichment analysis revealed that the common targets are primarily involved in processes such as the regulation of inflammatory responses, phosphorylation regulation, apoptosis signalling, and cellular responses to lipids and exogenous stimuli. KEGG enrichment analysis revealed that the relevant targets were primarily enriched in pathways such as TNF, *NF-κB*, IL-17, arachidonic acid metabolism, MAPK and PI3K-Akt. These findings provide a basis for subsequent validation studies focusing on gene expression associated with intestinal inflammation and COX-2 inhibitory activity (Figure 5).

### 3.3. The Effect of Xiasangju Processing Residues on the Production Performance and Organ Indices of Laying Hens

#### 3.3.1. Laying Hen Production Performance

The results of the feeding trial showed that the addition of Xiasangju processing residues to the diet had a significant effect on the laying rate and feed-to-egg ratio of laying hens (*p* < 0.05), but had no significant effect on average egg weight, the cracked, soft-shelled, and malformed egg rate, or average daily feed intake (*p* > 0.05). Among these, the XSJ-H group had the highest egg production rate, followed by the XSJ-L group; both were significantly higher than that of the XSJ-M group. The feed-to-egg ratio was higher in the XSJ-M group, whereas it was lower in the XSJ-H group (Table 4). The results suggest that Xiasangju processing residues, when included at appropriate levels, were associated with favorable production performance in laying hens during the late laying stage, with the 1.5% inclusion level showing relatively better performance under the conditions of this study.

#### 3.3.2. Laying Hen Organ Indices

The results of the organ index analysis (Table 5) showed that there were no significant differences in the liver, spleen, ovary and oviduct indices among the treatment groups (*p* > 0.05). Specifically, the liver index ranges from 1.47% to 1.61%, the spleen index from 0.07% to 0.08%, the ovary index from 0.31% to 0.37%, and the fallopian tube index from 3.24% to 3.74%. The results indicate that, at the levels of administration used in this study, Xiasangju processing residues did not cause any abnormal changes in the relative weights of the major immune, metabolic and reproductive organs.

### 3.4. The Effect of Xiasangju Processing Residues on Egg Quality and Serum Biochemical Parameters

#### 3.4.1. Egg Quality

The results of the egg quality assessment showed that Xiasangju processing residues had a significant effect on egg weight, shell strength, yolk colour, albumen height and the yolk index (*p* < 0.05), whilst having no significant effect on the egg shape index, yolk proportion or shell thickness (*p* > 0.05). In particular, the XSJ-H group exhibited higher egg weight and yolk colour, with both parameters showing a significant linear increase as the addition level rose; the XSJ-M group demonstrated higher eggshell strength, exhibiting a significant quadratic response; and the XSJ-L group showed relatively higher albumen height and yolk index. Although the Haugh units did not reach statistical significance, they showed a trend towards improvement (*p* = 0.074) (Table 6).

#### 3.4.2. Serum Biochemical Parameters

The serum biochemistry results showed that there were no significant differences (*p* > 0.05) in most indicators of immunoglobulins, mineral metabolism, liver function and lipid metabolism between the different treatment groups. No significant changes were observed in IgM, IgG, IgA, IP, Ca, ALT, AST, ALB, TC, TG, HDL-C and LDL-C, indicating that the Xiasangju processing residues did not cause any significant disruption to the immunoglobulin levels, mineral homeostasis or lipid metabolism in laying hens. There were significant differences in ALP and GLU levels between the groups (*p* < 0.05), with the 0.5% supplementation group showing higher ALP levels, and the 1.0% and 1.5% supplementation groups showing higher GLU levels than the control group. Given that there were no significant changes in ALT, AST and ALB, this study found no evidence that Xiasangju processing residues caused significant hepatocellular damage; however, the intergroup differences in ALP and GLU suggest that their potential effects on mineral metabolism, hepatobiliary enzyme activity or glucose metabolism require further investigation (Table 7).

### 3.5. The Effects of Xiasangju Processing Residues on Antioxidant Levels and Intestinal Morphology

#### 3.5.1. Antioxidant Levels in Egg Yolks and Liver

**Figure 6:**
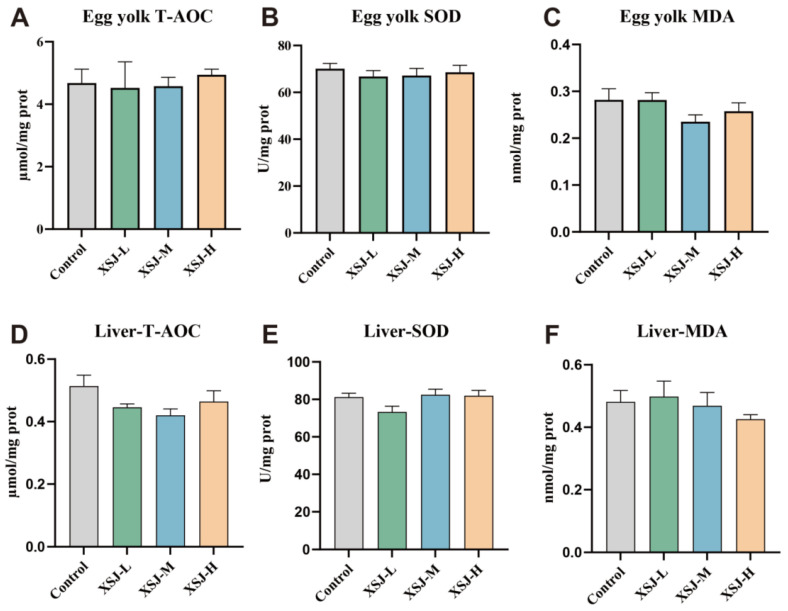
Effect of Xiasangju processing residues on antioxidant levels in laying hens. (**A**–**C**) Levels of T-AOC, SOD and MDA in egg yolk homogenates. (**D**–**F**) Levels of T-AOC, SOD and MDA in liver homogenates.

The results of antioxidant parameter analyses in egg yolk and liver (Figure 6A–F) showed that, in both egg yolk homogenates and liver homogenates, there were no significant differences (*p* > 0.05) in total antioxidant capacity (T-AOC), superoxide dismutase (SOD) activity, or malondialdehyde (MDA) content across the treatment groups. In particular, the levels of T-AOC, SOD and MDA in the egg yolk homogenate were fairly similar across the groups; similarly, no significant fluctuations were observed in the corresponding parameters in the liver homogenate.

#### 3.5.2. Intestinal Morphology

**Figure 7:**
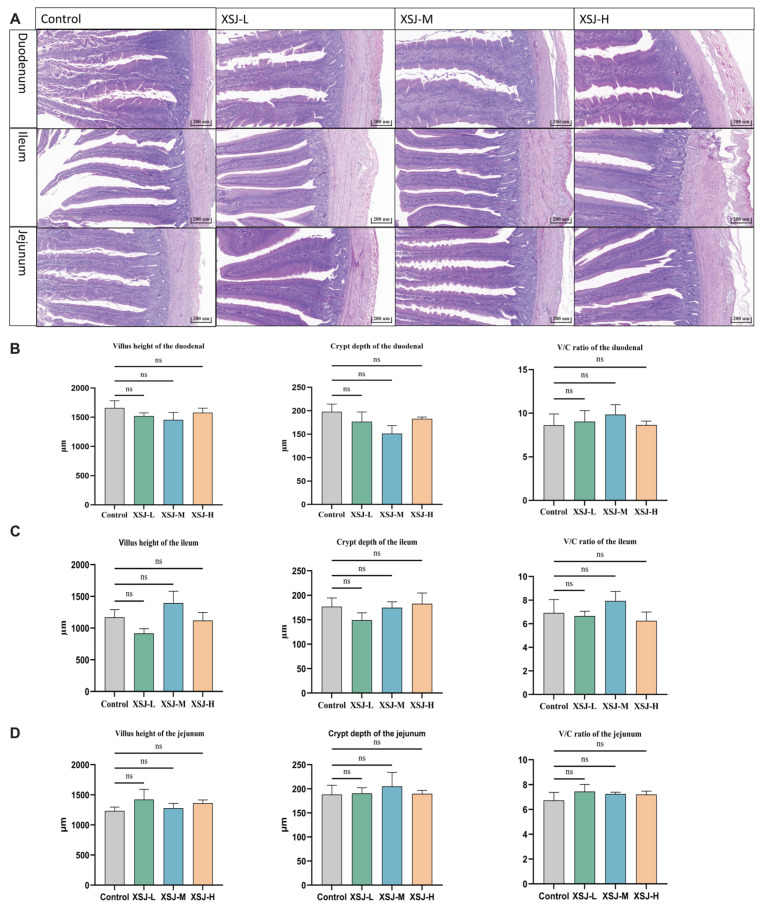
Effects of Xiasangju processing residues on the intestinal morphology of laying hens. (**A**) Representative histological images of the duodenum, ileum and jejunum. Scale bar = 200 μm. (**B**) Duodenal villus height, crypt depth and villus-to-crypt ratio. (**C**) Ileal villus height, crypt depth and villus-to-crypt ratio. (**D**) Jejunum villus height, crypt depth and villus-to-crypt ratio.

Morphological examination of the intestinal tissue revealed (Figure 7A) that the structures of the duodenum, jejunum and ileum were intact in all groups, with villi arranged in a relatively regular pattern; no obvious tissue damage was observed. Quantitative results indicate (Figure 7B–D) that Xiasangju processing residues had no significant effect on villus height, crypt depth or the villus-to-crypt ratio in the duodenum, jejunum and ileum (*p* > 0.05). This suggests that, at the levels of supplementation used in this study, Xiasangju processing residues were not associated with obvious disruption of the small intestinal mucosal structure or apparent intestinal morphological abnormalities.

### 3.6. The Effect of Xiasangju Processing Residues on the Expression of Genes Associated with Intestinal Inflammation

#### 3.6.1. Gene Expression Associated with Ileal Inflammation

**Figure 8:**
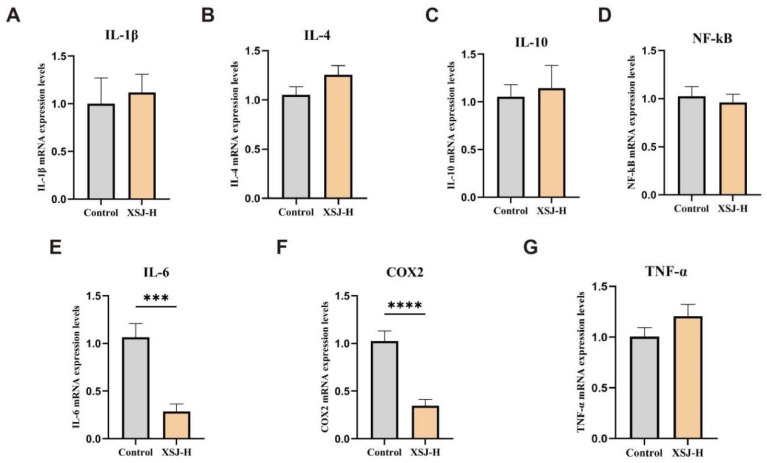
Effect of Xiasangju processing residues on the mRNA expression of genes associated with ileal inflammation in laying hens. (**A**–**G**) represent the relative expression levels of *IL-1β*, *IL-4*, *IL-10*, *NF-κB*, *IL-6*, *COX-2* and *TNF-α*, respectively. ns, not significant; *** *p* < 0.001, and **** *p* < 0.0001.

Based on the results regarding production performance and egg quality, the XSJ-H group and the Control group—which demonstrated superior overall performance—were selected for a comparative validation study of inflammatory gene expression and gut microbiota analysis. This design is intended to provide an initial exploration of potential mechanisms of action and is not intended to determine the full dose–response relationship for inflammatory and microbiota markers. qPCR results from ileal tissue showed that the XSJ-H group exhibited a significant downregulation of *IL-6* and *COX-2* mRNA expression (*p* < 0.001 or *p* < 0.0001), whereas the expression levels of *IL-1β*, *IL-4*, *IL-10*, *NF-κB* and *TNF-α* did not differ significantly from those in the Control group (*p* > 0.05) (Figure 8A–G). These results suggest that Xiasangju processing residue supplementation was associated with lower expression of selected inflammation-related genes in the ileum, with IL-6 and COX-2 showing more obvious changes in this comparison.

#### 3.6.2. Gene Expression Associated with Cecal Inflammation

**Figure 9:**
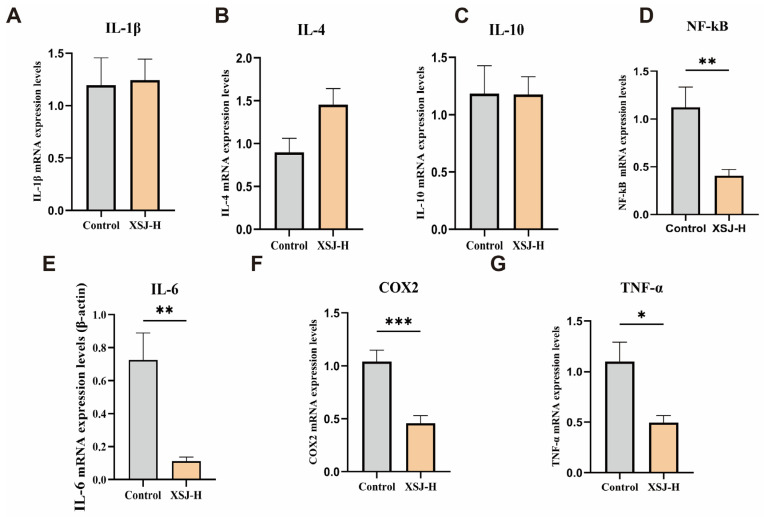
Effect of Xiasangju processing residues on the mRNA expression of genes associated with inflammation in the caecum of laying hens. (**A**–**G**) represent the relative expression levels of *IL-1β*, *IL-4*, *IL-10*, *NF-κB*, *IL-6*, *COX-2* and *TNF-α*, respectively. ns, not significant; * *p* < 0.05, ** *p* < 0.01, and *** *p* < 0.001.

In cecal tissue, the expression of *NF-κB*, *IL-6*, *COX-2* and *TNF-α* mRNA in the 1.5% treatment group was significantly lower than that in the control group; specifically, the differences for *NF-κB* and *IL-6* were extremely significant (*p* < 0.01), the difference for *COX-2* was extremely significant (*p* < 0.001), and the difference for *TNF-α* was significant (*p* < 0.05); No significant changes were observed in the expression of IL-1β, IL-4, or IL-10 (*p* > 0.05). Compared with the ileum, a broader range of inflammation-related genes showed lower expression in the caecum, suggesting that treatment-associated changes in local inflammatory markers may be more evident in the hindgut. (Figure 9A–G).

### 3.7. COX-2 Targeting by Active Components in Xiasangju Processing Residues and Their In Vitro Inhibitory Activity

#### 3.7.1. Docking of the Main Active Ingredient with the COX-2 Protein Molecule

**Figure 10:**
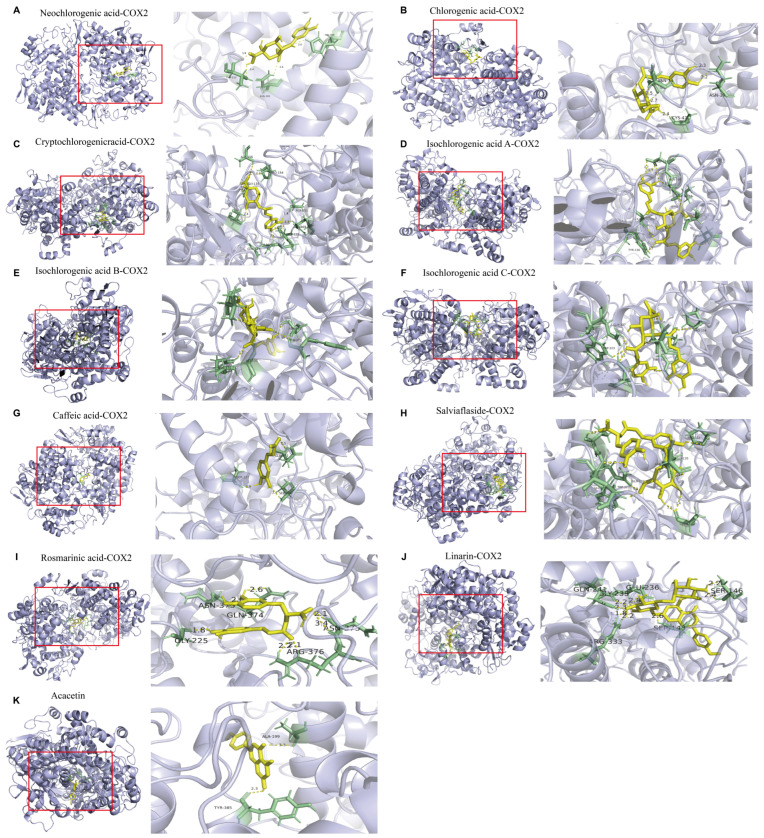
Molecular docking results of main active components in Xiasangju processing residue with COX-2 protein. (**A**–**K**) Molecular docking conformations of neochlorogenic acid, chlorogenic acid, cryptochlorogenic acid, isochlorogenic acid A, isochlorogenic acid B, isochlorogenic acid C, caffeic acid, Salviaflaside, Rosmarinic acid, Linarin and Acacetin with the COX-2 protein.The grey/purple cartoon structure represents the COX-2 protein; red rectangles indicate the enlarged docking regions; yellow sticks represent the docked ligands; green sticks indicate interacting amino acid residues; dashed lines and numbers indicate the predicted interaction distances.

Given that network pharmacology analysis suggests PTGS2 as a potential target and that COX-2 expression was lower in intestinal tissue of the XSJ-H group, this study further analysed the binding affinity of 11 major active components to the COX-2 protein. The molecular docking results show that the binding energies of all 11 compounds with COX-2 are below −6.0 kcal/mol, suggesting that they all possess a certain degree of binding potential (Figure 10A–K, Table 8). Among these, neochlorogenic acid, chlorogenic acid, Cryptochlorogenic acid and isochlorogenic acid A had lower binding energies of −10.6, −10.4, −10.3, and −10.1 kcal/mol, respectively, suggesting relatively strong predicted binding affinity for COX-2.

#### 3.7.2. In Vitro Inhibitory Effects of Xiasangju Processing Residues and Representative Active Components on COX-2 Activity

Residues from the processing of Xiasangju were used to evaluate the COX-2 inhibitory activity of the total extract; neochlorogenic acid was found to have the lowest binding energy in molecular docking with COX-2 among the 11 active compounds; linarin and rosmarinic acid were representative active compounds with relatively high concentrations in the preliminary quantitative analysis and were therefore included in subsequent in vitro functional validation.

**Figure 11:**
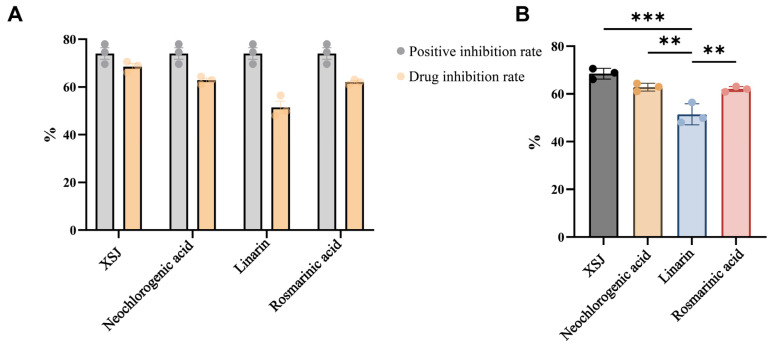
In vitro inhibitory effects of Xiasangju processing residue and its representative active components on COX-2 activity. (**A**) Comparison of COX-2 inhibition rates between Xiasangju processing residues, neochlorogenic acid, linarin and rosmarinic acid, and the positive control drug celecoxib. (**B**) Comparison of COX-2 inhibition rates among Xiasangju processing residues, neochlorogenic acid, linarin and rosmarinic acid. ns, not significant; ** *p* < 0.01, and *** *p* < 0.001.

The results of the COX-2 inhibitor screening showed that all four test compounds exhibited some COX-2 inhibitory activity (Figure 11A). Further comparison of the four test compounds (Figure 11B) revealed that the Xiasangju processing residue group exhibited the highest inhibition rate, which was significantly higher than that of the Linarin group (*p* < 0.001); the inhibition rate of the neochlorogenic acid group was also significantly higher than that of the Linarin group (*p* < 0.01); and the inhibition rate of the rosmarinic acid group was significantly higher than that of the Linarin group (*p* < 0.01).

There were no significant differences between the Xiasangju processing residues group and the neochlorogenic acid group or the rosmarinic acid group; nor were there any significant differences between the neochlorogenic acid group and the rosmarinic acid group. Overall, of the four test substances, linarin exhibited relatively weak COX-2 inhibitory activity, whereas the total extract of Xiasangju processing residues, neochlorogenic acid and rosmarinic acid all demonstrated high COX-2 inhibitory activity.

### 3.8. The Effect of Xiasangju Processing Residues on the Composition of the Gut Microbiota in Laying Hens

#### 3.8.1. Composition of the Foregut Microbiota

**Figure 12:**
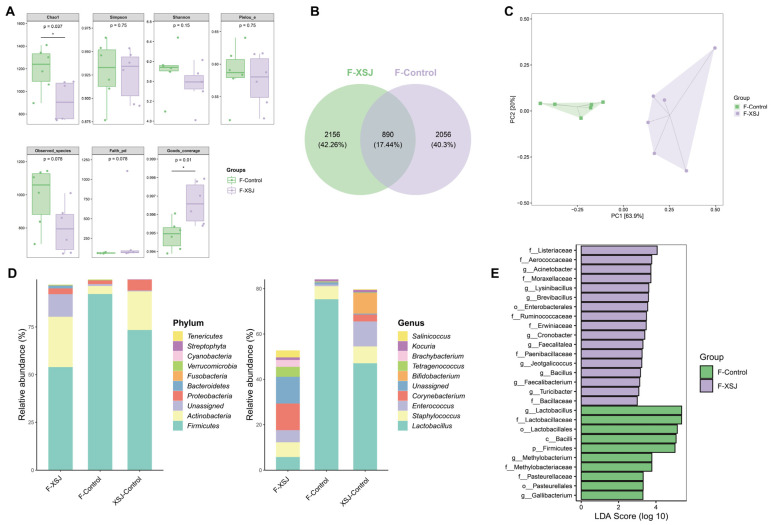
Effect of Xiasangju processing residues on the microbial community composition of the foregut in laying hens. (**A**) α-diversity indices (* *p* < 0.05). (**B**) Venn diagram of OTU distribution. (**C**) PCA analysis at the OTU level. (**D**) Relative abundance composition of the microbial community at the phylum and genus levels. (**E**) Differentially expressed microbial communities identified by LEfSe analysis and their LDA scores.

The results of 16S rRNA sequencing of the foregut microbiota revealed differences in microbial community structure between the 1.5% supplementation group and the control group. Analysis of α-diversity revealed that the Chao1 index in the 1.5% addition group was significantly lower than that in the control group (*p* < 0.05), whilst the observed species and Faith_pd indices showed a downward trend; however, there were no significant differences in the Shannon, Simpson and Pielou_e indices. This suggests that Xiasangju processing residue supplementation was mainly associated with changes in the richness of the foregut microbiota, with a relatively limited change in evenness (Figure 12A).

The Venn diagram shows differences in the composition of the two OTU groups (Figure 12B). PCA analysis revealed that the two groups of samples were clearly separated in the principal component space, suggesting an overall difference in the structure of the foregut microbiota (Figure 12C). At the phylum level, Firmicutes were the dominant phylum in both groups; however, in the 1.5% supplementation group, the relative abundance of Firmicutes decreased, whilst that of Actinobacteria and certain low-abundance phyla increased. At the genus level, the relative abundance of *Lactobacillus* decreased, while the abundances of various bacterial genera, including *Faecalibacterium*, *Bacteroides*, and *Bacillus*, changed (Figure 12D). LEfSe analysis further identified different bacterial communities between the two groups (Figure 12E).

#### 3.8.2. Composition of the Hindgut Microbiota

**Figure 13:**
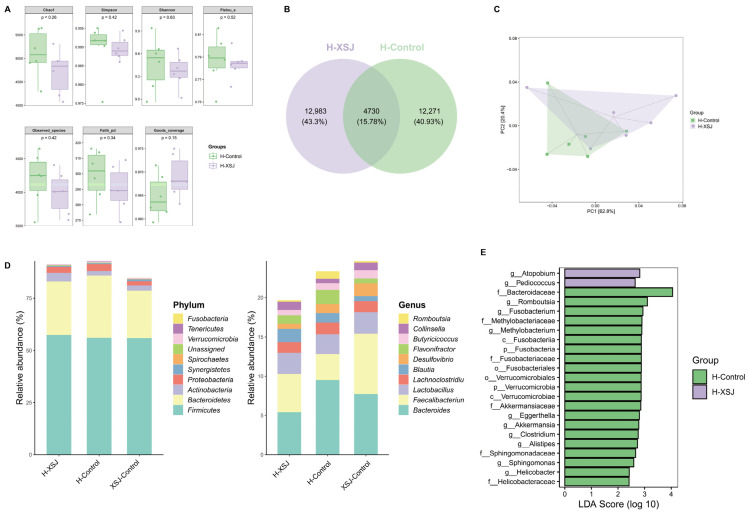
Effects of Xiasangju processing residues on the composition of the hindgut microbiota in laying hens. (**A**) α-diversity indices. (**B**) Venn diagram of OTU distribution. (**C**) OTU-level PCA analysis. (**D**) Relative abundance composition of microbial communities at the phylum and genus levels. (**E**) Differentially expressed microbial communities identified by LEfSe analysis and their LDA scores.

Analysis of the hindgut microbiota revealed that there were no significant differences in the α-diversity index between the 1.5% supplementation group and the control group (*p* > 0.05); however, the PCA results showed a certain degree of separation (Figure 13A–C). At the phylum level, the gut microbiota consists primarily of Firmicutes and Bacteroidetes; compared with the control group, the 1.5% supplementation group showed a slight increase in the relative abundance of Firmicutes and a slight decrease in the relative abundances of Bacteroidetes and Actinobacteria. At the genus level, the relative abundances of *Bacteroides*, *Romboutsia*, and *Lactobacillus* decreased, while genera such as *Atopobium* and *Pediococcus* were enriched in the 1.5% supplementation group (Figure 13D). LEfSe analysis further revealed that there were differences in taxonomic units between the two groups’ hindgut microbiota (Figure 13E). Overall, Xiasangju processing residues had a more pronounced effect on the richness and overall structure of the foregut microbiota, while their impact on the hindgut microbiota was primarily characterized by a trend toward compositional shifts, suggesting segment-specific responses of the gut microbiota to dietary Xiasangju processing residues.

## 4. Discussion

The results of this study show that, following industrial extraction, Xiasangju processing residues retain components such as linarin, rosmarinic acid, chlorogenic acids, caffeic acid and acacetin, whilst also containing measurable levels of crude protein, crude fat, crude fibre, minerals and amino acids. This suggests that these processing residues should not be regarded simply as industrial waste, but rather as plant-based raw materials that may retain residual bioactive compounds and basic nutritional value. Recent techno-economic and life-cycle evidence indicates that traditional Chinese medicine residues can be converted into value-added products, supporting the view that these by-products should be regarded as recyclable biomass resources rather than simple industrial waste [22].

Research into the application of natural plants and their extracts in the livestock industry has also shown that their functional value is generally linked to residual active ingredients, nutritional composition and the physiological state of the animals [23]. Previous studies have reported that Xiasangju residue can improve the gut health and microbial composition of weaned piglets [24]. Consequently, from the perspectives of resource utilisation and animal nutrition regulation, Xiasangju processing residues may warrant further evaluation as a potential feed ingredient for laying hens.

In this study, the addition of Xiasangju processing residues to the diet was associated with significant differences in egg production and the feed-to-egg ratio; the 1.5% supplementation group exhibited higher egg production and a lower feed-to-egg ratio, suggesting that this level of supplementation was associated with favorable production performance during the late laying period. In terms of egg quality, the 1.5% supplementation group exhibited higher egg weight and yolk colour, the 0.5% supplementation group performed better in terms of albumen height and yolk index, and the 1.0% supplementation group had higher eggshell strength. The optimal application rates vary across different indicators, suggesting that the effect of Xiasangju processing residues is not a simple linear dose–response relationship. Under conditions of oxidative stress, resveratrol can simultaneously improve laying hens’ production performance, egg quality and gut health [25]. Purslane extract has also been reported to improve growth performance, immuno-antioxidant status, intestinal barrier function, and gut microbiota composition in chickens challenged with *Escherichia coli*, supporting the potential of herb-derived extracts in poultry health regulation [26]. The effects of plant-based feed additives on the production performance, egg quality and small intestine morphology of laying hens are typically influenced by the additive dose and the characteristics of the base diet [27]. The role of rosemary supplementation in enhancing the production performance of laying hens and egg quality has also been supported by systematic evaluations [28]. These findings suggest that the favorable changes in production performance and certain egg quality parameters observed in this study may be associated with the combined contribution of various components, including residual phenolic acids, flavonoids, minerals and amino acids.

In terms of safety-related parameters, no significant differences were observed in the liver, spleen, ovary or fallopian tube indices across the treatment groups; furthermore, no marked abnormalities were observed in most serum immunoglobulin, mineral metabolism, liver function or lipid metabolism parameters. This suggests that, at the dose administered in this trial, Xiasangju processing residues were not associated with an obvious systemic burden based on the measured indicators. It should be noted that, whilst differences in ALP and GLU were observed between groups in this study, no significant changes were observed in ALT, AST or ALB, indicating that no clear evidence of hepatocellular damage has been observed. Antioxidant nutritional strategies, including vitamin E and polyphenol supplementation, have been reported to improve egg production, egg quality, and immune-related responses in aging hens [29]. At the same time, fermented plant by-products may also influence the gut health of older laying hens by improving the intestinal barrier and gut microbiota composition [30]. Consequently, further evaluation of the safety margins and stable conditions for the use of Xiasangju processing residues should continue, incorporating longer trial periods and finer dose gradients.

This study did not observe any significant effects of Xiasangju processing residues on T-AOC, SOD or MDA levels in egg yolks and livers, nor were there any significant changes in villus height, crypt depth or the villus-crypt ratio in the duodenum, jejunum and ileum. This suggests that, under normal feeding conditions, the primary effects of these processing residues may not manifest as enhanced systemic antioxidant capacity or morphological restructuring of the small intestine. In contrast, the 1.5% supplementation group showed a significant decrease in *IL-6* and *COX-2* expression in the ileum, with an even more pronounced reduction in *NF-κB*, *IL-6*, *COX-2* and *TNF-α* expression in the caecum. Gut health is recognised as a crucial foundation linking nutrient utilisation, immune homeostasis and production performance [31].

Chlorogenic acid derivatives can influence the inflammatory response of macrophages by modulating signals associated with inflammation and oxidative stress [32]. Rosmarinic acid has also been reported to exert anti-inflammatory effects by influencing NF-κB-related pathways [33]. Consequently, the observed responses in this study may be more closely associated with changes in local inflammatory markers in the gut, rather than with obvious improvements in small intestinal morphology or systemic antioxidant indicators.

The results of the network pharmacology analysis provide further insights into the aforementioned inflammatory regulation. A total of 173 potential target molecules were predicted for the 11 main active components; after intersecting these with targets associated with antioxidant and anti-inflammatory effects, 114 common targets were identified. PPI network screening revealed that targets such as *PTGS2*, *MMP9*, *SRC*, *EGFR* and *CASP3* occupy relatively important positions, whilst KEGG enrichment results implicated pathways including TNF, *NF-κB*, IL-17, arachidonic acid metabolism, MAPK and PI3K-Akt. As a plant-derived phenolic acid, chlorogenic acid has been reviewed as possessing multi-target anti-inflammatory and disease-modifying potential [34]. The antioxidant and anti-inflammatory properties of rosmarinic acid are also associated with the regulation of various inflammatory signalling pathways [35]. Acacetin has been reported to possess diverse bioactivities, including anti-inflammatory, anti-infective, and metabolic regulatory effects [36]. Further molecular docking studies suggested that all 11 active compounds had predicted binding potential with COX-2; in vitro COX-2 inhibition assays showed that the total extract of Xiasangju processing residues, neochlorogenic acid and rosmarinic acid displayed relatively high inhibitory activity under in vitro conditions. Previous studies have shown that acacetin can attenuate inflammation-related signalling by promoting the degradation of COX-2 [37]. It has also been reported that linarin reduces the expression of COX-2 and various inflammatory mediators by inhibiting the TLR4/NF-κB pathway [38]. These results suggest that the changes in COX-2-related inflammatory responses observed after Xiasangju processing residue supplementation may be associated with the combined contribution of various residual phenolic acids, flavonoids, and nutritional components. However, network pharmacology and molecular docking remain predictive in nature, and in vitro enzyme activity results do not fully reflect in vivo processes; further validation is required through studies involving tissue exposure, targeted metabolomics and pathway intervention trials.

The gut microbiota results suggest that Xiasangju processing residue supplementation may also be associated with changes in gut homeostasis through alterations in the microbial ecosystem. In the foregut, the Chao1 index decreased in the 1.5% supplementation group, and PCA results showed that the samples from the two groups were separated, indicating changes in the richness and overall structure of the foregut microbiota. This decrease in microbial richness should be interpreted cautiously, because a lower Chao1 index does not necessarily indicate an improvement in intestinal health. In the present study, the decrease in foregut richness may reflect a selective restructuring of the microbial community under dietary intervention rather than a uniformly beneficial effect. Therefore, the Chao1 result should be considered together with microbial composition, inflammatory gene expression, and other physiological indicators. Environmental and dietary stressors may impair intestinal integrity and microbial homeostasis in poultry, indicating that gut stability is an important target for nutritional intervention [39].

Probiotic supplementation has been reported to improve laying performance, egg quality, oxidative status, and gut health in laying hens, supporting microbiota-targeted nutritional strategies in layer production [40]. At the genus level, changes were observed in genera such as *Faecalibacterium*, *Turicibacter* and *Bacillus*. *Turicibacter* is associated with bile acid modification and host lipid metabolism [41]. The genus’s tolerance to bile salts in the ecological niche of the chicken’s small intestine also suggests that it may have a segment-specific functional significance [42]. In the hindgut, no significant changes were observed in α-diversity, but alterations were noted in genera such as *Bacteroides*, *Romboutsia*, *Lactobacillus*, *Atopobium* and *Pediococcus*. The clearer PCA separation in the foregut than in the hindgut further suggests that the microbiota-modulating effect of Xiasangju processing residues may be more evident in the foregut, indicating a segment-specific response along the intestinal tract. Recent evidence also indicates that *Bacillus* subtilis supplementation can improve production performance, egg quality, serum parameters, and intestinal health in laying hens [43]. Consequently, the changes in the microbial community observed in this study are best interpreted as a restructuring of the gut microbiota, rather than simply being attributed to an increase in probiotics or a suppression of harmful bacteria. Overall, Xiasangju processing residues, when added at a level of 1.5%, were associated with favorable changes in indicators such as egg production rate, egg weight and yolk colour, and were not associated with obvious abnormalities in major organ indices or most serum biochemical parameters. The observed responses may involve changes in local intestinal inflammatory markers and gut microbiota composition, with COX-2-related pathways serving as a possible link, rather than being explained primarily by enhanced systemic antioxidant activity or improvements in small intestinal morphology. It should be noted that the mechanistic validation in this study focused primarily on the Control group and the XSJ-H group; it does not yet fully elucidate the dose–response relationship of inflammatory and microbiota markers at different supplementation levels. Furthermore, the microbiota analysis was limited mainly to community composition and differential microbiota, and did not simultaneously measure microbial metabolites such as short-chain fatty acids and bile acids.

A major limitation of the present study is that complete formulations and calculated nutrient compositions of the final experimental diets were not available. Although the basal diet and the inclusion levels of Xiasangju processing residues were recorded, the experimental diets were not documented as fully isocaloric, isonitrogenous, or balanced for amino acids, calcium, and phosphorus. Xiasangju processing residues contain not only residual phytochemicals but also nutritional components, including crude protein, crude fat, dietary fibre, minerals, and amino acids. Therefore, the improvements observed in laying performance, inflammatory gene expression, and gut microbiota composition may reflect the combined contribution of nutritional components and residual bioactive compounds. The current experimental design does not allow complete separation of nutritional effects from phytochemical effects. Accordingly, the proposed roles of linarin, rosmarinic acid, chlorogenic acids, acacetin, COX-2-related inflammatory regulation, and gut microbiota modulation should be interpreted as potential mechanisms rather than definitive causal evidence.

The identification of the value of by-products from the production of traditional Chinese medicinal materials and their resource-efficient utilisation still requires the establishment of clearer quality control and functional evaluation systems, including the monitoring of representative residual active compounds such as linarin and rosmarinic acid. The high-value utilization of Chinese medicine processing residues should not be limited to waste reduction, but should also be based on clear characterization of residual bioactive compounds, functional value, and application safety [44]. Future research could incorporate nutritionally matched diets, complete dietary formulation records, a more comprehensive dose gradient, target metabolomics, and gut metabolite analysis to further distinguish nutritional effects from phytochemical effects and elucidate the mechanisms underlying the effects of Xiasangju processing residues on the production performance and gut health of laying hens in the late laying phase.

## 5. Conclusions

This study shows that, following industrial extraction, Xiasangju processing residues retain active components such as linarin, rosmarinic acid, chlorogenic acids, caffeic acid, and acacetin, and contain certain amounts of crude protein, crude fiber, minerals, and amino acids. Dietary supplementation with Xiasangju processing by-products was associated with favorable changes in selected parameters performance and egg quality parameters in laying hens during the late laying period. Under the conditions of this study, the 1.5% supplementation group showed relatively favorable results in terms of egg production rate, feed-to-egg ratio, egg weight, and yolk color, without obvious abnormalities in major organ indices or most serum biochemical parameters. Further results suggest that these responses may be associated with COX-2-related inflammatory regulation and changes in gut microbiota composition. However, because complete nutrient-balanced formulations of the final experimental diets were not available, the present study cannot fully distinguish nutritional effects from phytochemical effects. Overall, Xiasangju processing residues may have potential as a dietary supplement for laying hens in the late laying phase; however, the optimal inclusion rate, in vivo exposure to active components, nutritional contribution, and specific mechanisms require further validation using nutritionally matched diets and purified active compounds.

## Figures and Tables

**Table 1:** Composition and Nutritional Levels of the Base Diet (on an air-dry basis) %.

Ingredients	Content (%)	Nutrition Levels	Content (%)
Corn	55	ME/(MJ/kg)	11.48
Soybean meal	25.1	CP	15.773
Bran	4	EE	2.56
CaHPO_4_	1.50	Lys	0.76
Limestone	9	Met	0.35
Coarse stone grain	2.40	Ca	3.65
Minerals premix	3	TP	0.59
Total	100	AP	0.37

The premix is a compound premix that provides vitamins and trace minerals per kilogram of diet; its specific composition is formulated according to the manufacturer’s recommended inclusion rate.

**Table 2:** Content of 11 Major Active Compounds in Xiasangju Processing Residues.

Ingredients	Content (μg·g^−1^)
Neochlorogenic acid	3.86 ± 1.79
Cryptochlorogenic acid	3.84 ± 3.01
Chlorogenic acid	4.25 ± 2.02
Caffeic acid	9.08 ± 4.75
Isochlorogenic acid B	1.60 ± 1.01
Isochlorogenic acid A	2.38 ± 1.21
Isochlorogenic acid C	3.98 ± 1.48
Rosmarinic acid	25.92 ± 13.47
Salviaflaside	10.68 ± 4.71
Linarin	83.22 ± 5.62
Acacetin	10.18 ± 5.53

Data are presented as mean ± standard deviation; ND indicates not detected; the full results for all batches of samples and QC samples are given in Supplementary Table S4.

**Table 3:** Conventional nutritional composition of Xiasangju processing residues.

Indicators	Content
Crude protein (%)	12.60 ± 1.49
Gross energy (MJ·kg^−1^)	18.39 ± 1.85
Moisture (%)	6.64 ± 0.34
Crude ash (%)	13.17 ± 1.58
Crude fat (%)	7.52 ± 1.28
Dietary fibre (%)	12.49 ± 1.52
Calcium (%)	1.86 ± 0.90
Phosphorus (%)	1.81 ± 0.36
Total amino acids (%)	8.45

Total amino acids refer to the sum of the 17 standard amino acids; details of the 17 amino acids are provided in Supplementary Table S5.

**Table 4:** Effects of Xiasangju processing residues on production performance of laying hens.

Items	Control	XSJ-L	XSJ-M	XSJ-H	*p*-Value	Linear	Quadratic
Laying rate (%)	89.62 ^ab^ ± 0.69	90.66 ^a^ ± 0.43	87.45 ^b^ ± 1.12	91.27 ^a^ ± 0.82	0.015	0.926	0.098
Average egg weight (g)	61.54 ± 0.64	61.58 ± 0.41	61.48 ± 0.51	62.37 ± 0.28	0.522	0.594	0.601
Cracked, soft-shelled, and malformed egg rate (%)	0.18 ± 0.05	0.23 ± 0.08	0.25 ± 0.06	0.18 ± 0.06	0.811	0.950	0.351
ADFI (g)	113.50 ± 0.53	113.04 ± 0.36	113.70 ± 1.38	113.06 ± 0.09	0.906	0.774	0.484
Feed/egg	2.06 ^b^ ± 0.02	2.02 ^b^ ± 0.04	2.12 ^a^ ± 0.07	1.99 ^b^ ± 0.08	0.008	0.875	0.542

Different lowercase letters within the same row indicate a significant difference (*p* < 0.05). The same applies throughout. Cracked, soft-shelled, and malformed egg rate refers to the proportion of cracked eggs, soft-shelled eggs, and malformed eggs among the total number of eggs laid.

**Table 5:** Effects of Xiasangju processing residues on organ indices in laying hens.

Items	Control	XSJ-L	XSJ-M	XSJ-H	*p*-Value	Linear	Quadratic
Liver indices (%)	1.61 ± 0.10	1.47 ± 0.11	1.47 ± 0.01	1.56 ± 0.13	0.711	0.761	0.268
Spleen Index (%)	0.07 ± 0.00	0.08 ± 0.00	0.08 ± 0.01	0.07 ± 0.03	0.728	0.870	0.280
Ovarian Index (%)	0.34 ± 0.02	0.31 ± 0.04	0.37 ± 0.04	0.33 ± 0.02	0.543	0.987	0.816
Fallopian Tube Index (%)	3.24 ± 0.17	3.74 ± 0.26	3.24 ± 0.50	3.35 ± 0.14	0.209	0.830	0.305

Data are presented as mean ± standard error.

**Table 6:** Effect of Xiasangju processing residues on egg quality.

Items	Control	XSJ-L	XSJ-M	XSJ-H	*p*-Value	Linear	Quadratic
Egg weight (g)	59.43 ^c^ ± 0.71	61.44 ^bc^ ± 0.83	61.27 ^bc^ ± 1.23	62.76 ^ab^ ± 0.59	0.041	0.024	0.735
Egg shape index (%)	73.24 ± 0.60	75.93 ± 0.86	75.39 ± 0.88	74.71 ± 1.99	0.101	0.271	0.037
Eggshell strength (kgf)	2.99 ^b^ ± 0.14	3.46 ^a^ ± 0.21	3.76 ^ab^ ± 0.20	3.33 ^ab^ ± 0.15	0.039	0.108	0.019
Egg yolk color	4.37 ^b^ ± 0.18	4.81 ^ab^ ± 0.21	4.56 ^b^ ± 0.24	5.37 ^a^ ± 0.21	0.013	0.007	0.381
Haugh unit	65.63 ± 2.49	73.63 ± 3.47	68.23 ± 2.79	70.51 ± 1.58	0.074	0.337	0.188
Albumen height (mm)	6.70 ^b^ ± 0.26	7.59 ^a^ ± 0.14	7.23 ^ab^ ± 0.23	7.34 ^a^ ± 0.15	0.031	0.097	0.067
Yolk index	0.35 ^b^ ± 0.01	0.41 ^a^ ± 0.02	0.37 ^ab^ ± 0.01	0.37 ^ab^ ± 0.01	0.007	0.317	0.011
Yolk percentage	0.28 ± 0.01	0.29 ± 0.02	0.29 ± 0.01	0.29 ± 0.01	0.729	0.274	0.805
Eggshell thickness (mm)	0.30 ± 0.01	0.29 ± 0.01	0.30 ± 0.01	0.31 ± 0.01	0.801	0.352	0.384

Data are presented as mean ± standard error; values within the same row with different superscript lowercase letters indicate significant differences (*p* < 0.05).

**Table 7:** Effects of Xiasangju processing residues on the serum biochemistry of laying hens.

Item	Control	XSJ-L	XSJ-M	XSJ-H	*p*-Value
IgM (g/L)	0.0137 ± 0.0042	0.0263 ± 0.0096	0.0175 ± 0.0025	0.0271 ± 0.0029	0.290
IgG (g/L)	0.0175 ± 0.0041	0.0150 ± 0.0033	0.0100 ± 0.0033	0.0063 ± 0.0026	0.105
IgA (g/L)	0.0014 ± 0.0014	0.0075 ± 0.0075	0.0013 ± 0.0013	0.0050 ± 0.0027	0.677
IP (mmol/L)	2.88 ± 0.21	2.47 ± 0.14	2.96 ± 0.45	2.83 ± 0.23	0.457
Ca (mmol/L)	5.63 ± 0.32	5.37 ± 0.23	5.50 ± 0.07	5.26 ± 0.22	0.690
ALT (U/L)	11.38 ± 2.97	9.05 ± 2.33	6.32 ± 2.38	8.62 ± 2.90	0.597
AST (U/L)	279.73 ± 16.33	277.76 ± 13.94	304.76 ± 13.31	305.11 ± 18.45	0.436
ALP (U/L)	288.62 ^b^ ± 54.07	636.24 ^a^ ± 117.01	526.71 ^ab^ ± 85.84	340.75 ^b^ ± 51.67	0.023
ALB (g/L)	25.21 ± 1.35	32.87 ± 7.41	26.87 ± 0.53	26.68 ± 0.86	0.506
GLU (mmol/L)	11.56 ^b^ ± 0.27	11.94 ^ab^ ± 0.21	12.44 ^a^ ± 0.26	12.63 ^a^ ± 0.35	0.040
TC (mmol/L)	4.20 ± 0.58	3.40 ± 0.18	3.78 ± 0.17	3.73 ± 0.22	0.452
TG (mmol/L)	20.23 ± 2.10	19.47 ± 1.73	30.93 ± 9.29	21.52 ± 1.84	0.290
HDL-C (mmol/L)	1.69 ± 0.30	1.57 ± 0.10	1.68 ± 0.07	1.63 ± 0.08	0.954
LDL-C (mmol/L)	1.46 ± 0.36	1.06 ± 0.07	1.09 ± 0.04	1.06 ± 0.07	0.367

Data are expressed as mean ± standard error. Values within the same row with different superscript lowercase letters indicate significant differences between groups (*p* < 0.05). IgM: immunoglobulin M; IgG: immunoglobulin G; IgA: immunoglobulin A; ALT: alanine aminotransferase; AST: aspartate aminotransferase; ALP: alkaline phosphatase; ALB: albumin; GLU: glucose; TC: total cholesterol; TG: triglycerides; HDL-C: high-density lipoprotein cholesterol; LDL-C: low-density lipoprotein cholesterol.

**Table 8:** Docking of major active components of Xiasangju processing residues with COX-2 protein.

Name	COX-2 Binding Energy (kcal/mol)
Neochlorogenic acid	−10.6
Chlorogenic acid	−10.4
Cryptochlorogenic acid	−10.3
Isochlorogenic acid A	−10.1
Isochlorogenic acid B	−9.2
Isochlorogenic acid C	−8.7
Caffeic acid	−8.6
Salviaflaside	−8.4
Rosmarinic acid	−8.3
Linarin	−8.1
Acacetin	−6.5

## Data Availability

The data supporting the findings of this study are available within the article. The raw sequencing data have been deposited in the NCBI Sequence Read Archive (SRA) under BioProject accession number PRJNA997628.

## Supplementary Materials

The following supporting information can be downloaded at: https://www.mdpi.com/article/10.3390/ani16121841/s1. Table S1: Reported chemical constituents of *Prunella vulgaris* L., *Morus alba* L. and *Chrysanthemum indicum* L.; Table S2: Standard curves, linear equations, and correlation coefficients of 11 active compounds in Xiasangju processing residue; Table S3: LC-MS method validation results; Table S4: Contents of 11 active compounds in different batches of Xiasangju processing residue; Table S5: Contents of common amino acids in Xiasangju processing residue; Figure S1: Two-dimensional molecular structures of the main active components in Xiasangju processing residue.
