# Supplementary material for: Xiasangju Processing Residues Improve Production Performance and Modulate Intestinal Inflammation and Gut Microbiota in Laying Hens
Source: Animals (Basel). 2026 Jun 15;16(12):1841. doi: 10.3390/ani16121841 (PMC13295601; doi:10.3390/ani16121841)

Supplementary Table S1. Reported chemical constituents of Prunella vulgaris L., Morus alba L. and Chrysanthemum indicum L.

English name	Molecular formula	Chinese name	Source	Chemical class
Caffeic acid	C9H8O4	Caffeic acid	Prunella vulgaris L.	Phenolic acids
chrysophanol	C15H10O4	Chrysophanol	Prunella vulgaris L.	Anthraquinones
Rosmarinic acid	C18H16O8	Rosmarinic acid	Prunella vulgaris L.	Phenolic acids
Salviaflaside	C24H26O13	Salviaflaside	Prunella vulgaris L.	Phenolic acids
Salicylic acid	C7H6O3	Salicylic acid	Prunella vulgaris L.	Phenolic acids
α-spinasterol	C29H48O	α-Spinasterol	Prunella vulgaris L.	Steroids
2-hydroxy-3-methyl anthraquinone	C15H10O3	2-Hydroxy-3-methy lanthraquinone	Prunella vulgaris L.	Anthraquinones
Cynaroside	C21H20O11	Cynaroside	Prunella vulgaris L.	Flavonoids
Danshensu	C9H10O5	Danshensu	Prunella vulgaris L.	Phenolic acids
Ethyl 3-(3,4-dihydroxyphenyl)acrylate	C11H12O4	Ethyl caffeate	Prunella vulgaris L.	Phenolic acids
Hesperidin	C28H34O15	Hesperidin	Prunella vulgaris L.	Flavonoids
Isoorientin	C21H20O11	Isoorientin	Prunella vulgaris L.	Flavonoids
Kaempferol	C15H10O6	Kaempferol	Prunella vulgaris L.	Flavonoids
Luteolin	C15H10O6	Luteolin	Prunella vulgaris L.	Flavonoids
Methyl oleanolate	C32H52O3	Methyl oleanolate	Prunella vulgaris L.	Oleanane-type triterpenoids
Methyl rosmarinate	C19H18O8	Methyl rosmarinate	Prunella vulgaris L.	Phenolic acids
Niga-ichigoside F1	C36H58O11	Niga-ichigoside F1	Prunella vulgaris L.	Oleanane-type triterpenoids
Oleanic acid	C30H48O3	Oleanolic acid	Prunella vulgaris L.	Oleanane-type triterpenoids
Quercetin	C15H10O7	Quercetin	Prunella vulgaris L.	Flavonoids

rhein	$C15H8O6$	Rhein	Prunella vulgaris L.	Anthraquinones
Rutin	$C27H30O16$	Rutin	Prunella vulgaris L.	Flavonoids
stigmasterol	$C29H48O$	Stigmasterol	Prunella vulgaris L.	Steroids
tanshinone I	$C18H12O3$	Tanshinone I	Prunella vulgaris L.	Anthraquinones
Ursolic Acid	$C30H48O3$	Ursolic acid	Prunella vulgaris L.	Ursane-type triterpenoids
Wogonin	$C16H12O5$	Wogonin	Prunella vulgaris L.	Flavonoids
β-amyrin	$C30H50O$	β-Amyrin	Prunella vulgaris L.	Oleanane-type triterpenoids
benzoic acid	$C7H6O2$	Benzoic acid	Prunella vulgaris L.	Phenolic acids
cis-4-coumaric acid	$C9H8O3$	p-Coumaric acid	Prunella vulgaris L.	Phenolic acids
(E)-Ferulic acid	$C10H10O4$	Ferulic acid	Prunella vulgaris L.	Phenolic acids
Syringic acid	$C9H10O5$	Syringic acid	Prunella vulgaris L.	Phenolic acids
Cinnamic acid	$C9H8O2$	Cinnamic acid	Prunella vulgaris L.	Phenolic acids
Sinapinic acid	$C11H12O5$	Sinapinic acid	Prunella vulgaris L.	Phenolic acids
Rhynchophylline	$C22H28N2O4$	Rhynchophylline	Prunella vulgaris L.	Alkaloids

Supplementary Table S2. Standard curves, linear equations, and correlation coefficients of 11 active compounds in Xiasangju processing residue

Compound name	Linear equation	R^2
Caffeic acid	y=617x+23861	0.9995
Salviaflaside	y=382x-6742	0.9990
Rosmarinic acid	y=500x-11277	0.9984
Neochlorogenic acid	y=422x+7696	0.9993
Cryptochlorogenic acid	y=428x+7197	0.9994
Chlorogenic acid	y=422x+7696	0.9994
Isochlorogenic acid B	y=427x-10180	0.9994
Isochlorogenic acid A	y=342x-4715	0.9991
Isochlorogenic acid C	y=401x-16398	0.9987
Linarin	y=664x+4369	0.9995
Acacetin	y=1610x-18029	0.9980

Supplementary Table S3. LC-MS method validation results

Compound	Repeatability RSD/%	Precision RSD/%	Stability RSD/%
Salviaflaside	1.95	1.2	0.72
Linarin	3.15	1.04	4.87
Acacetin	3.12	1.59	6.81
Neochlorogenic acid	2.15	1.83	2.61
Cryptochlorogenic acid	2.91	1.83	1.97
Chlorogenic acid	1.99	1.29	1.95
Caffeic acid	1.46	1.07	3.42
Isochlorogenic acid B	3.65	2.85	2.29
Isochlorogenic acid A	3.95	4.65	6.76
Isochlorogenic acid C	4.89	2.55	4.36
Rosmarinic acid	0.63	0.93	1.1

Supplementary Table S4. Contents of 11 active compounds in different batches of Xiasangju processing residue.

Sample name	Neochlorogenic acid	Cryptochlorogenic acid	Chlorogenic acid	Caffeic acid	Isochlorogenic acid B	Isochlorogenic acid A	Isochlorogenic acid C	Rosmarinic acid	Salviaflaside	Linarin	Acacetin
Sample-1	1.73	13.83	8.30	1.99	ND	1.72	3.23	17.28	11.15	49.19	11.41
Sample-2	ND	ND	ND	2.06	ND	1.02	2.42	6.05	3.54	69.25	12.32
Sample-3	ND	ND	ND	2.01	ND	1.06	2.40	6.27	3.91	69.44	12.16
Sample-4	1.79	1.08	1.78	5.69	ND	1.41	3.01	11.27	6.44	54.71	5.29
Sample-5	4.79	5.57	5.59	6.99	4.13	6.32	9.06	15.70	6.74	263.83	15.09
Sample-6	3.85	3.04	4.12	7.03	1.41	2.80	4.50	24.88	5.01	179.87	7.93
Sample-7	1.41	1.19	1.53	5.24	0.49	1.89	3.69	12.12	6.81	101.08	9.42
Sample-8	2.76	1.64	2.33	10.24	ND	1.75	3.28	21.05	9.35	44.89	2.95
Sam	7.38	5.72	7.36	16.5	2.08	3.13	4.68	45.28	13.5	59.	2.5

ple-9				7					9	58	6
Sample-10	3.81	2.55	4.01	11.97	0.58	2.20	3.51	30.00	13.03	60.16	7.39
Sample-11	6.24	4.34	5.92	14.24	1.59	3.22	4.44	51.09	17.28	48.41	5.50
Sample-12	4.09	3.24	3.97	9.45	1.18	2.54	4.30	34.46	11.39	84.62	8.74
Sample-13	2.91	2.13	2.71	7.74	ND	1.77	3.25	22.14	9.66	61.50	4.06
Sample-14	1.32	2.49	1.19	6.37	ND	1.25	2.73	20.56	11.33	27.05	14.60
Sample-15	4.01	2.45	4.27	14.78	ND	2.47	3.63	43.35	20.91	46.11	25.58
QC-1	5.15	4.00	4.95	12.83	1.52	2.74	4.48	34.81	14.07	93.93	12.98
QC-2	5.26	3.99	4.93	13.83	1.49	2.74	4.52	35.32	13.96	91.28	12.51
QC-3	5.29	4.13	4.98	14.35	1.52	2.84	4.46	34.92	14.02	93.02	12.72
Mean (n = 18)	3.86±1.79	3.84±3.01	4.25±2.02	9.08±4.75	1.6±1.01	2.38±1.21	3.98±1.48	25.92±13.47	10.68±4.71	83.22±56.2	10.18±5.53

ND: Not detected; the concentration was not calculated because it was below the limit of quantification.

Supplementary Table S5. Contents of common amino acids in Xiasangju processing residue

Nutritional component	Content (%)	Nutritional component	Content (%)
Aspartic acid (ASP)	0.88	Valine (VAL)	0.54
Glutamic acid (GLU)	1.29	Methionine (MET)	0.17
Serine (SER)	0.40	Phenylalanine (PHE)	0.53
Histidine (HIS)	0.24	Isoleucine (ILE)	0.44
Glycine (GLY)	0.51	Leucine (LEU)	0.78
Threonine (THR)	0.42	Lysine (LYS)	0.40
Arginine (ARG)	0.48	Proline (PRO)	0.49
Alanine (ALA)	0.54	Cysteine (CYS)	0.07
Tyrosine (TYR)	0.27		

Total	8.45

Supplementary Figure S1. Two-dimensional molecular structures of the main active components in Xiasangju processing residue

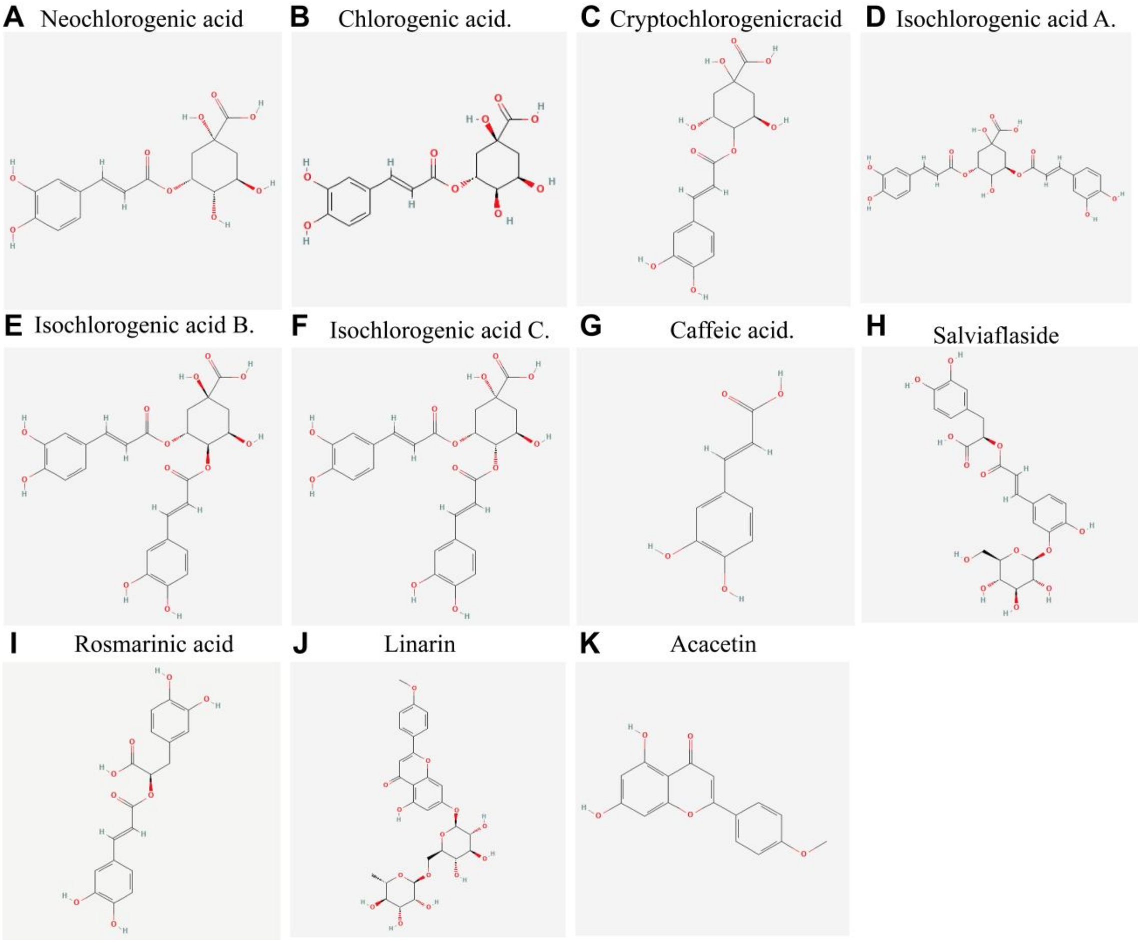

Supplement: Supplementary file 1 [file animals-16-01841-s001.zip › animals-4368374-supplementary.pdf]
